# The Prevalence of Idiopathic or Inherited Isolated Dystonia: A Systematic Review and Meta‐Analysis

**DOI:** 10.1002/mdc3.13524

**Published:** 2022-08-24

**Authors:** Alex Medina, Christelle Nilles, Davide Martino, Catherine Pelletier, Tamara Pringsheim

**Affiliations:** ^1^ Department of Clinical Neurosciences, Cumming School of Medicine University of Calgary Calgary Alberta Canada; ^2^ Hotchkiss Brain Institute University of Calgary Calgary Alberta Canada; ^3^ Public Health Agency of Canada Ottawa Ontario Canada; ^4^ Department of Psychiatry, Pediatrics, Community Health Sciences University of Calgary Calgary Alberta Canada

**Keywords:** dystonia, incidence studies, prevalence studies, blepharospasm

## Abstract

**Background:**

A systematic review of epidemiological studies of primary dystonia from 1985 and 2010 found an overall prevalence of 16.43 per 100,000 (95% CI = 12.09–22.32).

**Methods:**

We performed a systematic review of studies from 2010 and 2022 to determine if there are important differences in epidemiology between these time periods.

**Results:**

Nineteen studies were included. Incidence of cervical dystonia, blepharospasm, and oromandibular dystonia were each reported in one study; one study reported incidence for all adult onset idiopathic focal dystonias combined. Using data from 11 studies, we performed random effects meta‐analyses of the prevalence of cervical dystonia (9.95 per 100,000; 95% CI = 3.51–28.17), blepharospasm (2.82 per 100,000; 95% CI = 1.12–7.12), laryngeal dystonia (0.40 per 100,000; 95% CI = 0.09–1.83), upper limb dystonia (1.27 per 100,000; 95% CI = 0.36–4.52), oromandibular dystonia (0.57 per 100,000; 95% CI = 0.15–2.15), and idiopathic or inherited isolated dystonia all subtypes combined (30.85 per 100,000; 95% CI = 5.06–187.74). All studies reported more cases of dystonia in females. There was no significant difference in prevalence by subgroup analysis based on time of study publication (1985–2010 vs. 2010–2022). Subgroup analysis of differences in prevalence by dystonia subtype by continent using all studies published (1985–2022) revealed significant regional differences in the prevalence of cervical and laryngeal dystonia.

**Conclusion:**

The incidence and prevalence of idiopathic or inherited isolated dystonia in the last decade was not significantly different from earlier reports. Population‐based studies across multiple geographic areas are needed to obtain a clearer understanding of the epidemiology of this condition.

The Consensus Committee on the phenomenology and classification of dystonia defines dystonia as a movement disorder characterized by sustained or intermittent muscle contractions causing abnormal, often repetitive movements, postures, or both.^1^ Dystonic movements are typically patterned, twisting, and may be tremulous. The classification of dystonia has changed over time, reflecting an increased understanding of the clinical manifestations and etiology of dystonia. Previous classification systems for dystonia distinguished primary (autosomal dominant or other genetic causes) and secondary dystonia syndromes, including dystonia‐plus and degenerative, complex, and acquired forms.^2^, ^3^ The most recent classification scheme was published in 2013.^1^ According to this classification, dystonia is classified by Axis I Clinical Characteristics, and Axis II Etiology. Axis I is subdivided into clinical characteristics of dystonia and associated features. Clinical characteristics of dystonia include age of onset, body distribution (ie, focal, segmental, multifocal, generalized, and hemidystonia), and temporal pattern. Associated features include if the dystonia is isolated or combined with another movement disorder and if there are any co‐occurring neurological or systemic manifestations. Axis II Etiology specifies if there is nervous system pathology (ie, evidence of degeneration or structural lesion) or an inherited or acquired cause.

A previous systematic review and meta‐analysis on the incidence and prevalence of primary dystonia identified 16 original studies published between 1985 and 2010, one on incidence and 15 on prevalence.^4^ The overall prevalence of primary dystonia was 16.43 per 100,000 (95% CI = 12.09–22.32) from 12 service‐based studies, with higher estimates reported from three population‐based studies. Adult‐onset focal forms of primary dystonia were more common than primary generalized dystonia, with the most prevalent forms being cervical dystonia (4.98 per 100,000; 95% CI = 3.58–6.94) and blepharospasm (4.24 per 100,000; 95% CI = 2.92–6.18). Since the publication of the previous review, several additional studies on the incidence and prevalence of dystonia have been published, with some dystonia experts concluding that previous prevalence estimates may have been low because of under ascertainment of cases.^5^ In this manuscript, we have performed a systematic review and meta‐analysis of studies of dystonia incidence and prevalence published between 2010 and 2022, following similar methodology used for the 1985 to 2010 review, to determine if there are important differences in epidemiology between these two time periods. Similar to our previous review, we focused on primary dystonia, or what is classified in the current terminology as idiopathic or inherited forms of isolated dystonia.

## Methods

A systematic review and meta‐analysis were performed following a predetermined protocol submitted and registered with PROSPERO (CRD42021234966). The protocol followed the standards recommended in the Preferred Reporting Items for Systematic Reviews and Meta‐Analysis guidelines.^6^ The authors confirm that the approval of an institutional review board was not required for this work. The authors confirm that patient consent was not required for this work. We confirm that we have read the Journal's position on issues involved in ethical publication and affirm that this work is consistent with those guidelines.

### Search Strategy

The literature search was developed and peer‐reviewed by a health science librarian. The search strategy was applied to two electronic databases, MEDLINE and EMBASE. The period was limited to studies published between January 2010 and March 2021; the search was restricted to studies published in English or French. We overlapped the search strategy period of the previous and current systematic review by 1 year to improve detection of all relevant studies. We used controlled vocabulary for dystonia, prevalence and incidence. Subheadings for epidemiology, etiology, diagnosis, and classifications were included. In addition, the text word (tw) and keyword (kw) searches were added to broaden our search to incorporate pre‐indexing studies. The articles identified in each database were combined in Endnote X9 and subsequently uploaded to COVIDENCE platform for duplicate removal, screening, and extraction stages. Review articles on the topic were additionally explored to identify studies not captured through our search. The complete search strategy parameters for the two databases can be found in Appendix S1.

### Study Screening and Selection

All the resulting citations and abstracts were uploaded to the COVIDENCE platform and evaluated separately by two authors (including A.M., C.N., or T.P.). As a first step, all the titles and abstracts exploring the prevalence or incidence of idiopathic or inherited forms of isolated dystonia in a specific geographical region or population were identified. Subsequently, a full‐text review of the selected studies was conducted. Discordant selections in the previous two steps were automatically detected by the COVIDENCE platform, and they were separated for re‐evaluation to reach a consensus. The exclusion criteria at this stage were: conference abstracts; duplicates; prevalence or incidence not reported; not in English or French; insufficient or unclear fragmented data for analysis. Inter‐rater reliability was calculated in COVIDENCE using Cohen's κ statistic.

### Data Extraction from Selected Studies

Data extraction was performed using a standardized data collection table, one for incidence and one for prevalence studies. The variables included were: first author, year of publication, country, population size, number of cases, data source used to identify cases, diagnostic criteria used, incidence/prevalence date, calculated incidence/prevalence, and subgroup calculated incidence/prevalence. We only included unique, non‐overlapping samples to avoid multiple publication biases. After a cross‐check process (by A.M., C.N., and T.P.), a consolidated entry form was subsequently developed.

### Risk of Bias

A quality assessment was performed for each study based on criteria developed from standards for evaluating prevalence studies.^7^, ^8^ Each study was given a score out of 8 based on the quality of the study methodology and the sample's representativeness.

### Data Synthesis and Analyses

The included studies were classified according to whether they examined the incidence or prevalence of idiopathic or inherited forms of dystonia. An estimated pooled prevalence by dystonia subtype for studies published between 2010 and 2022 was calculated using a random effects model with Comprehensive Meta‐Analysis software version 3.3.070. The raw data provided in each publication on the population size studied and the number of cases detected were entered into the meta‐analysis. If the population size studied or number of cases detected were not provided for a study, we used the reported standardized incidence or prevalence (ie, cases per 100,000) to back calculate the population size or number of cases, as required. Heterogeneity in pooled estimates was assessed using the *I* ^2^ and the Cochran *Q* statistics.

We performed sub analyses to determine if there were significant differences in the prevalence of dystonia by subtype based on (1) the time period the study was published, that is 1985 to 2010 versus 2010 to 2022, and (2) the continent the study was performed in, including all studies published from 1985 to 2022.

## Results

The electronic database search was executed on March 18, 2021. A total of 2647 citations were identified, 611 in MEDLINE and 2036 in EMBASE; 388 duplicates were automatically detected and eliminated in the COVIDENCE platform, resulting in 2259 studies. Through title and abstract screening, 2212 citations were excluded; Cohen's κ at this stage was 0.71. In the full‐text review, 47 studies were examined for eligibility, 25 were subsequently excluded. The main reasons for exclusion at this stage were: conference abstracts (n = 15), wrong study design or outcomes (n = 6, ie, studies that described clinical features of patients with dystonia without reporting prevalence or incidence), duplicates not filtered through COVIDENCE (n = 2) and articles not written in English or French (n = 2). The 22 studies selected for data extraction were further scrutinized, and five studies were excluded for the following reasons: not reporting incidence or prevalence (n = 1), review article (n = 1) and duplicate data reporting (n = 3). The Cohen's κ was of 0.79. A total of 17 studies were selected for qualitative synthesis, of which two reported incidence rates and 15 examined the prevalence (see Fig. S1). We re‐executed the search on January 26, 2022 to find articles published after March 18, 2021. A total of 262 additional abstracts were retrieved, with two additional articles meeting our inclusion criteria, both reporting on dystonia incidence and prevalence.

### Risk of Bias

Only two studies implemented a door‐to‐door detection method to estimate incidence or prevalence, graded with 5 and 3 points of 8.^9^, ^10^ One study used a probability sampling method and was graded with 5 points.^11^ The remaining studies used health service‐based methodology and received grades between 2 and 4 points. (Table S1)

### Incidence

Four studies examined the incidence of adult onset idiopathic or inherited isolated dystonia, one from the United States,^12^ one from Taiwan,^13^ one from Japan,^14^ and one from Wales.^15^ All studies reported more incident cases of dystonia in females compared to males. LaHue et al^12^ explored the incidence of cervical dystonia in the United States, in northern California, over a period of 5 years. The overall incidence rate for cervical dystonia was 1.18 per 100,000 person‐years (95% CI = 0.35–2.0). A subgroup incidence was reported for gender, age and racial categories, finding a higher incidence in females (1.81 per 100,000 person‐years), higher incidence within the age group of 60 to 69 (2.57 per 100,000 person‐years), and a higher incidence rate in Caucasians (1.56 per 100,000 person‐years). Sun et al^13^ evaluated the incidence of blepharospasm in Taiwan over a period of 14 years. The mean annual incidence of blepharospasm reported was 10 per 100,000 person‐years. A higher incidence was identified in the age group comprising 50 to 59 (19 per 100,000 person‐years), and in females (12 per 100,000 person‐years). Bailey et al^15^ evaluated the incidence of adult‐onset idiopathic dystonia in Wales over a period of 24 years. The mean annual incidence was 87.6 per 100,000 person‐years, with 63% of new diagnoses occurring in females. Only case counts (rather than cases per 100,000 per year) were provided for dystonia subtypes. Through back‐calculation, using 47,557,077 person years of observation as the denominator, the incidence of cervical dystonia was 55.85 per 100,000 person years, blepharospasm was 2.48 per 100,000 person years, orofacial dystonia was 0.05 per 100,000 person years, and upper limb was 0.09 per 100,000 person years. Yoshida^14^ studied the incidence of oromandibular dystonia in Kyoto, Japan, over a period of 5 years and reported a mean annual incidence of 1.2 per 100,000 person‐years (95% CI = 0.68–1.9) for idiopathic oromandibular dystonia. (Table S2).

### Prevalence

Seventeen studies explored the prevalence of idiopathic or inherited isolated forms of dystonia with geographical representation across 12 countries and 1 territory (Brazil, Cameroon, China, Colombia, Egypt, Finland, Faroe Islands (Denmark), Ireland, Japan, Sweden, Thailand, United States, and Wales). Eleven studies provided data that could be included in the meta‐analysis, with adequate data to perform analyses by dystonia subtype. Because of poor consistency between studies in the reporting of sex and age groups of cases and study populations, meta‐analysis of prevalence by sex and by age group could not be performed. Six studies were not included in the meta‐analysis (but remain in Table S3) for the following reasons. One study^16^ used a log‐linear model to predict the total number of dystonia cases, but did not provide data on the actual number of cases detected and we were, therefore, unable to perform back calculations. Two studies^10^, ^17^ did not specify whether the cases ascertained had isolated idiopathic/inherited or secondary forms of dystonia. One study explored the prevalence of DYT1 mutation carriers.^18^ Two studies were not included in the meta‐analysis as they evaluated the prevalence of dystonia within specific patient populations rather than the general population. One study of these studies^19^ was conducted in a dedicated Orofacial Pain and Dental Sleep Medicine Clinic at the University of Minnesota. The authors identified six cases of oromandibular dystonia within a defined population of people with temporomandibular disorders, resulting in a prevalence of 170 cases per 100,000. Finally, one study was a population‐based study of tremor, with a multi‐step case ascertainment process.^11^ In study participants identified as having tremor in stage I, the number of participants with isolated dystonia of the neck or upper limb was identified in stage II.

#### Cervical Dystonia

Seven studies reported the prevalence of cervical dystonia^20^, ^21^, ^22^, ^23^, ^24^, ^25^, ^26^ yielding a pooled prevalence of 9.95 cases per 100,000 (95% CI = 3.51–28.17; *I* ^2^ = 4%; *Q* = 6.3) (Table 1).

**TABLE 1:** Prevalence of cervical dystonia, blepharospasm, and laryngeal dystonia 2010 to 2022

Location	Study	Cases	Sample	Prevalence per 100,000	95% CI
Cervical dystonia					
South America (Brazil)	Bezerra et al^20^	51	1,483,715	3.44	2.61–4.52
Asia (China)	Wang et al^21^	416	54,938,000	0.76	0.69–0.83
Asia (Thailand)	Bhidayasiri et al^22^	99	1,039,595	9.52	7.82–11.6
Europe (Sweden)	Hellberg et al^23^	1742	9,640,000	18.07	17.24–18.94
Europe (Faroe Islands)	Joensen^24^	23	48,100	47.82	31.78–71.95
Europe (Finland)	Ortiz et al^25^	589	1,580,758	37.26	34.37–40.39
Europe (Ireland)	Williams et al^26^	410	3,325,821	12.33	11.19–13.58
Total *Q* = 6.3, *I* ^2^ = 4				9.95	3.51–28.17
Blepharospasm					
South America (Brazil)	Bezerra et al^20^	60	1,483,715	4.04	3.14–5.21
Asia (China)	Wang et al^21^	640	54,938,000	1.16	1.08–1.26
Asia (Thailand)	Bhidayasiri et al^22^	12	1,039,595	1.15	0.66–2.03
Asia (China)	Fang et al^27^	338	14,498,400	2.33	2.1–2.59
Europe (Sweden)	Hellberg et al^23^	1133	9,640,000	11.75	11.09–12.46
Europe (Finland)	Ortiz et al^25^	47	1,580,758	2.97	2.23–3.96
Europe (Ireland)	Williams et al^26^	102	3,325,821	3.07	2.52–3.72
Total *Q* = 2.4 *I* ^2^ = 0				2.82	1.12–7.12
Laryngeal dystonia					
South America (Brazil)	Bezerra et al^20^	10	1,483,715	0.67	0.36–1.25
Asia (China)	Wang et al^21^	7	54,938,000	0.01	0.01–0.03
Europe (Faroe Islands)	Joensen^24^	1	48,100	2.08	0.29–14.76
Europe (Finland)	Ortiz et al^25^	22	1,580,758	1.39	0.92–2.11
Europe (Ireland)	Williams et al^26^	18	3,325,821	0.54	0.34–0.86
Total *Q* = 5.4 *I* ^2^ = 26				0.40	0.09–1.83

#### Blepharospasm

The prevalence of blepharospasm was examined in seven studies.^20^, ^21^, ^22^, ^23^, ^25^, ^26^, ^27^ The overall pooled prevalence was 2.82 cases per 100,000 (95% CI = 1.12–7.12; *I* ^2^ = 0%; *Q* = 2.4) (Table 1).

#### Laryngeal Dystonia

Prevalence rates for laryngeal dystonia were explored in five studies,^20^, ^21^, ^24^, ^25^, ^26^ resulting in a pooled prevalence of 0.40 cases per 100,000 (95% CI = 0.09–1.83; *I* ^2^ = 26%; *Q* = 5.4) (Table 1).

#### Upper Limb Dystonia

Prevalence rates for upper limb dystonia were reported in six studies,^20^, ^21^, ^22^, ^24^, ^25^, ^26^ resulting in a pooled prevalence of 1.27 cases per 100,000 (95% CI = 0.36–4.52; *I* ^2^ = 0%; *Q* = 5) (Table 2).

**TABLE 2:** Prevalence of upper limb dystonia, oromandibular dystonia, all dystonia subtypes 2010 to 2022

Location	Study	Cases	Sample	Prevalence per 100,000	95% CI
Upper limb dystonia					
South America (Brazil)	Bezerra et al^20^	28	1,483,715	1.89	1.3–2.73
Asia (China)	Wang et al^21^	38	54,938,000	0.07	0.05–0.1
Asia (Thailand)	Bhidayasiri et al^22^	21	1,039,595	2.02	1.32–3.1
Europe (Faroe Islands)	Joensen^24^	4	48,100	8.32	3.12–22.16
Europe (Finland)	Ortiz et al^25^	31	1,580,758	1.96	1.38–2.79
Europe (Ireland)	Williams et al^26^	39	3,325,821	1.17	0.86–1.61
Total *Q* = 5.0 *I* ^2^ = 0				1.27	0.36–4.52
Oromandibular dystonia					
South America (Brazil)	Bezerra et al^20^	3	1,483,715	0.20	0.07–0.63
Asia (China)	Wang et al^21^	33	54,938,000	0.06	0.04–0.08
Asia (Japan)	Yoshida^14^	84	1,465,701	5.73	4.63–7.10
Europe (Finland)	Ortiz et al^25^	9	1,580,758	0.57	0.30–1.09
Europe (Ireland)	Williams et al^28^	6	3,325,821	0.18	0.08–0.40
Europe (Sweden)	Hellberg et al^23^	140	9,640,000	1.45	1.23–1.71
Europe (Faroe Islands)	Joensen^24^	1	48,100	2.08	0.29–14.76
Total *Q* = 4.8 *I* ^2^ = 0				0.57	0.15–2.15
All dystonia subtypes combined					
Africa (Egypt)	Badry et al^9^	3	33,285	9.01	2.91–27.94
South America (Brazil)	Bezerra et al^20^	227	1,483,715	15.30	13.43–17.42
Asia (China)	Wang et al^21^	1481	54,938,000	2.70	2.56–2.84
Asia (Thailand)	Bhidayasiri et al^22^	141	1,039,595	13.56	11.50–16.00
Europe (Wales)	Bailey et al^15^	32,662	2,721,833	1200.00	1187.13–1213.00
Europe (Sweden)	Hellberg et al^23^	4239	9,640,000	43.97	42.67–45.32
Europe (Faroe Islands)	Joensen^24^	29	48,100	60.3	41.9–86.8
Europe (Finland)	Ortiz et al^25^	1316	1,580,758	83.3	78.9–87.9
Europe (Ireland)	Williams et al^26^	592	3,325,821	17.80	16.42–19.29
Total *Q* = 3.1, *I* ^2^ = 0				30.85	5.06–187.74

#### Oromandibular Dystonia

The prevalence of oromandibular dystonia was examined in seven studies^14^, ^20^, ^21^, ^22^, ^23^, ^25^, ^26^ resulting in a pooled prevalence of 0.57 cases per 100,000 (95% CI = 0.15–2.15; *I* ^2^ = 0%; *Q* = 4.8) (Table 2).

#### Idiopathic or Inherited Isolated Dystonia—All Subtypes Combined

Nine studies reported the prevalence of all subtypes of idiopathic or inherited isolated dystonia^9^, ^15^, ^20^, ^21^, ^22^, ^23^, ^24^, ^25^, ^26^ with a combined prevalence of 30.85 cases per 100,000 (95% CI = 5.06–187.74; *I* ^2^ = 0%; *Q* = 3.1) (Table 2). All studies reported a greater number of prevalent cases in women relative to men.

### Subanalyses

#### By Time Period of Study Publication, 1985 to 2010 Versus 2010 to 2022

We performed a dedicated sub‐analysis of dystonia prevalence by subtype based on the time period of study publication, 1985 to 2010, versus 2010 to 2022 (see Table 3). There was no significant difference in dystonia prevalence by subtype based on the time period of study publication. Studies published between 2010 and 2022 had higher prevalence of cervical dystonia and all dystonia subtypes combined; lower prevalence of blepharospasm and laryngeal dystonia; and similar prevalence of upper limb and oromandibular dystonia to studies published between 1985 to 2010.

**TABLE 3:** Dystonia prevalence per 100,000, sub analysis by time period of study publication

Dystonia subtype	1985–2010	2010–2022	Heterogeneity and *P* value
Cervical dystonia	4.98, 95% CI = 3.58, 6.94 *I* ^2^ = 34.5 *Q* = 12.2	9.95, 95% CI = 3.51, 28.17 *I* ^2^ = 4, *Q* = 6.3	*Q* = 1.5, *P* = 0.22
Blepharospasm	4.24, 95% CI = 2.92, 6.18 *I* ^2^ = 8.5, *Q* = 9.8	2.82, 95% CI = 1.12, 7.12 *I* ^2^ = 0, *Q* = 2.4	*Q* = 0.6, *P* = 0.42
Laryngeal dystonia	1.54, 95% CI = 0.65, 3.61 *I* ^2^ = 0, *Q* = 3.6	0.40, 95% CI = 0.09, 1.83 *I* ^2^ = 26, *Q* = 5.4	*Q* = 2.3, *P* = 0.13
Upper limb dystonia	2.23, 95% CI = 1.31, 3.80 *I* ^2^ = 0.9, *Q* = 6.1	1.27, 95% CI = 0.36, 4.52 *I* ^2^ = 0, *Q* = 5.0	*Q* = 0.6, *P* = 0.42
Oromandibular dystonia	0.51, 95% CI = 0.17, 1.53 *I* ^2^ = 0, *Q* = 4.2	0.57, 95% CI = 0.15, 2.15 *I* ^2^ = 0, *Q* = 4.8	*Q* = 0.01, *P* = 0.90
All dystonia subtypes combined	16.42, 95% CI = 12.10, 22.31 *I* ^2^ = 46.6, *Q* = 7.5	30.85, 95% CI = 5.06, 187.74 *I* ^2^ = 0, *Q* = 3.1	*Q* = 0.6, *P* = 0.46

#### By Continent, 1985 to 2022

As there was no significant difference in prevalence by time period, we pooled all studies published from 1985 to 2022 to have greater statistical power to perform dedicated sub analyses of dystonia prevalence by subtype based on the continent the study was performed in (see Table 4, and Tables S4–S9 for individual study details). We detected statistically significant differences in the prevalence of cervical dystonia and laryngeal dystonia by continent.

**TABLE 4:** Dystonia prevalence per 100,000, sub analysis by continent, and all studies 1985 to 2022

Dystonia subtype	Europe	Asia	South America	Africa	Heterogeneity and *P* value
Cervical dystonia	11.38 95% CI = 7.35, 17.61 *I* ^2^ = 30.1, *Q* = 12.9	2.50 95% CI = 0.71,8.75 *I* ^2^ = 0, Q1.6	3.43 95% CI = 2.61, 4.52 *I* ^2^ = 0, *Q* = 0	No data	*Q* = 21.7, *P* < 0.0001
Blepharospasm	4.14 95% CI = 2.51–6.82 *I* ^2^ = 0, *Q* = 5.7	2.59 95% CI = 1.19, 5.66 *I* ^2^ = 4.3, *Q* = 4.2	4.04 95% CI = 3.14, 5.21 *I* ^2^ = 0, *Q* = 0	No data	*Q* = 1.18, *P* = 0.55
Laryngeal dystonia	1.34 95% CI = 0.73, 2.45 *I* ^2^ = 0, *Q* = 6.3	0.01 95% CI = 0.006, 0.03 *I* ^2^ = 0, *Q* = 0	0.67 95% CI = 0.36, 1.25 *I* ^2^ = 0, *Q* = 0	No data	*Q* = 99.9, *P* < 0.0001
Upper limb dystonia	2.39 95% CI = 1.43, 4.01 *I* ^2^ = 23.8, *Q* = 7.9	1.01 95% CI = 0.20, 5.17 *I* ^2^ = 0, *Q* = 2.9	1.89 95% CI = 1.30, 2.73 *I* ^2^ = 0, *Q* = 0	No data	*Q* = 1.22, *P* = 0.54
Oromandibular dystonia	0.58 95% CI = 0.25,1.33 *I* ^2^ = 10.0, *Q* = 7.8	0.58 95% CI = 0.04, 9.08 *I* ^2^ = 0, *Q* = 1.4	0.20 95% CI = 0.07, 0.63 *I* ^2^ = 0, *Q* = 0	No data	*Q* = 2.2, *P* = 0.33
All dystonia subtypes combined	50.87 95% CI = 10.27, 251.56 *I* ^2^ = 0, *Q* = 2.7	8.63 95% CI = 3.05, 24.43 *I* ^2^ = 0, *Q* = 1.7	19.48 95% CI = 17.36,21.86 *I* ^2^ = 0, *Q* = 0	9.01 95% CI = 2.91,27.94 *I* ^2^ = 0, *Q* = 0	*Q* = 5.5, *P* = 0.14

## Discussion

In this systematic review and meta‐analysis, we aimed to update estimates of the epidemiology of idiopathic or inherited isolated dystonia. Although it was not possible to provide pooled estimates of the incidence of dystonia because of the lack of data, there were sufficient data to provide an updated meta‐analysis of the prevalence of dystonia, including overall prevalence and prevalence by body region affected. From studies published between 2010 and 2022, the overall prevalence estimate for dystonia of 30.85 per 100,000 was higher than the 16.42 per 100,000 reported by studies published from 1985 to 2010,^4^ but a subgroup analysis did not reveal statistically significant changes in prevalence estimates by time period. We observed significant differences in the pooled prevalence of cervical and laryngeal dystonia by continent when subanalyses of all published studies were performed, with studies performed in Europe having a significantly higher prevalence of these disorders. These differences observed by continent could be the result of variations in health service provision and care seeking by region, expertise in diagnosis of the condition, and genetic factors. We used a random effects model for all our analyses based on significant clinical heterogeneity in the methodology used between included studies. This model is the only appropriate way to apprehend the heterogeneity of the studies and to be able to generalize the results to the general population.

The methodology used across studies may directly influence prevalence estimates. One would expect that door‐to‐door studies or studies using random population sampling would provide the least biased estimates of dystonia prevalence, as long as such studies use standardized criteria for dystonia diagnosis and classification, and include examination of every participant by a skilled clinician, because of the known diagnostic difficulties associated with this disorder.^28^ Unfortunately, this remains an under‐used strategy for dystonia prevalence studies. Only two studies on the prevalence of multiple neurological disorders (including dystonia) using a door‐to‐door survey were conducted between 2010 and 2021, neither of which provided detailed data on dystonia diagnosis or subtype. In our previous systematic review and meta‐analysis,^4^ the included population‐based studies provided higher overall prevalence estimates for dystonia than the service‐based studies, likely because of the detection of undiagnosed or untreated cases. Opinion leaders in the epidemiology of dystonia^5^ are calling for population‐based studies of adult‐onset dystonia and provide recommendations on sample size for such studies based on hypothesized prevalence. Population‐based door‐to‐door studies, however, require substantial financial and human resources and are confined to specific geographic areas, limiting generalizability of findings.

The predominantly service‐based studies included in our analysis are likely affected by selection bias. Data derived primarily from tertiary movement disorders clinics or other medical specialties make it very likely that mild, untreated, or undiagnosed cases are systematically excluded from prevalence estimates. Studies using administrative codes as a case ascertainment method are limited, as they may incorporate combined forms or even drug‐induced forms of dystonia. The risk of bias assessment method we used, which awards higher scores to population‐based studies, resulted in the majority of included studies receiving low scores because of their case ascertainment methods. Even within service‐based studies using similar methodology on the same continent, prevalence estimates differed more than 10‐fold.^15^, ^25^

Looking specifically at the four studies of dystonia incidence, all four studies used a service‐based methodology, with Bailey et al^15^ studying multiple subtypes of adult‐onset idiopathic dystonia, and the other three studies looking at one specific dystonia subtype each. Bailey et al^15^ performed a retrospective, population‐based study using anonymized electronic data from community health care and hospital records. The methodology for estimating the incidence of dystonia followed a case‐ascertainment algorithm. A list of codes was used to identify dystonia cases, using codes relevant to dystonia diagnosis, symptoms, and therapy, which was reviewed by a neurologist with movement disorder expertise. Codes lists were created to maximize the positive predictive value. Using the anonymized records of 90 patients with a confirmed diagnosis of adult‐onset idiopathic focal dystonia as the reference population, the algorithm had a sensitivity of 79%. The specificity of the algorithm is not reported, and it is therefore, uncertain what percentage of ascertained cases had a definite dystonia diagnosis. Bailey et al^15^ reported a much higher incidence of cervical dystonia, 55.85 per 100,000 person years, compared to LaHue et al,^12^ who reported an incidence of 1.18 per 100,000 person years. LaHue et al's^12^ study using data from Kaiser Permanente, implemented a three‐step case identification procedure to precisely identify patients with cervical dystonia. This included identification of possible cases through diagnostic codes used in inpatient, outpatient, and pharmacy records, review of all health service utilization used by those identified with a cervical dystonia diagnosis, and finally extensive review by a movement disorders expert, including a neurological examination or review of standardized videotape data collection, thereby minimizing the inclusion of false positives. In contrast, the Bailey et al^15^ study reported a lower incidence of blepharospasm of 2.48 per 100,000 person years, compared to Sun et al,^13^ who reported an incidence of 10 per 100,000 person years. Sun et al^13^ used the Longitudinal Health Insurance Database in Taiwan and defined incident cases of blepharospasm as persons who were diagnosed at least once with blepharospasm at an outpatient or inpatient visit between 2000 and 2013, in whom no possible secondary causes were identified. No case validation procedure was performed. The final incidence study of dystonia was performed by a single expert clinician who personally identified every person in Kyoto diagnosed with oromandibular dystonia over a defined period,^14^ eliciting an incidence of 1.2 per 100,000 person‐years, compared to Bailey et al's^15^ estimate of 0.05 per 100,000 person years. These disparate results, with Bailey et al^15^ finding a much higher rate of cervical dystonia, and lower rates of blepharospasm and oromandibular dystonia than the other incidence studies, suggests (1) potentially important racial differences in dystonia incidence, (2) that cultural factors may influence care seeking for dystonia, or (3) that the service‐based approach to epidemiological surveillance is truly inadequate to obtain accurate measurements of incidence.

The case definition for dystonia across studies was subject to different nomenclatures or criteria. We believe that the impact of these differences on incidence and prevalence estimates to be minimal, as the studies that provided data for our synthesis gave clear descriptions of the various forms of dystonia included in their case counts. The updated classification system primarily entails a syndromic approach, mainly relying on phenomenology,^29^ to facilitate communication across clinicians and researchers. Nonetheless, multiple challenges have been detected, including the complexity of its applicability. Furthermore, the genotype–phenotype correlation has been growing primarily through exome‐wide sequencing, and many causative variants have been identified for previously considered isolated focal dystonia phenotypes.^30^ Sasikumar et al^31^ identified that the new classification system was not adopted in 8.9% of studies investigating different aspects or dystonia. In addition, over 30% implemented a combined terminology. Albanese et al^32^ recently proposed parameters on how to extrapolate the previous terminology with the new classification system. Similarly, in our study, we identified few studies incorporating the new updated system.

Our study bears multiple limitations. First, there are still only a few studies on the epidemiology of idiopathic or inherited isolated dystonia, with most existing studies reporting data on the most common adult‐onset focal forms. Most of the studies were conducted in specialized clinics, which very likely underestimate the incidence and prevalence estimates for this condition because of under‐recognition of this neurological disorder. In addition, the subgroup analysis was limited to geography, as demographic factors were underreported or not standardized across studies. Even this subgroup analysis was limited; there were insufficient data to perform analyses by latitude, which has been suggested as an important risk factor for blepharospasm.^33^ Methodological discrepancies between epidemiological studies pose a major barrier to obtaining accurate estimates of the incidence and prevalence of idiopathic or inherited isolated dystonia. Service‐based studies within referral centers may also be biased because dystonia patients may be assessed and treated in other clinical settings (eg, blepharospasm and oromandibular dystonia may be followed in ophthalmologic or dental centers), resulting in further under ascertainment of cases. Although a synthesis of results was not possible with subgrouping based on age or sex, the studies included in our review suggest that there are significant demographic differences, with all studies reporting a higher number of incident and prevalent cases of idiopathic or inherited isolated dystonia in women than men and increasing prevalence with age.

In conclusion, our systematic review and meta‐analysis on the incidence and prevalence of idiopathic or inherited isolated dystonia did not find evidence of statistically significant changes in the epidemiology of this condition from studies published in the last decade compared to earlier studies. This underscores the a need for population‐based studies across multiple geographic areas to obtain a clearer understanding on the epidemiology of this condition and how it varies based on age, sex, and ethnicity.

## Author Roles

(1) Research Project: A. Conception, B. Organization, C. Execution; (2) Statistical Analysis: A. Design, B. Execution, C. Review and Critique; (3) Manuscript Preparation: A. Writing of the First Draft, B. Review and Critique.

A.M.: 1C, 2B, 3A

D.M.: 3B

C.P.: 1A, 2A, 3B

C.N.: 1C, 2B, 3A

T.P.: 1A, 1B, 1C, 2A, 2B, 2C, 3A, 3B

## Disclosures

**Ethical Compliance Statement:** The authors confirm that the approval of an institutional review board was not required for this work. The authors confirm that patient consent was not required for this work. We confirm that we have read the Journal's position on issues involved in ethical publication and affirm that this work is consistent with those guidelines.

**Funding Source and Conflicts of Interest:** This study was funded by the Public Health Agency of Canada. The authors declare that there are no conflicts of interest relevant to this work.

**Financial Disclosures for the Previous 12 Months:** D.M. has received compensation for consultancies for Sunovion and Merz Pharmaceuticals, honoraria from Movement Disorders Society, Dystonia Medical Research Foundation Canada and Merz Pharmaceuticals, royalties from Springer‐Verlag, research support from Ipsen Corporate, funding grants from Dystonia Medical Research Foundation Canada, Parkinson Canada, The Owerko Foundation, and the Michael P. Smith Family. A.M. and C.N. have none to report. T.P. has received research funding from the Public Health Agency of Canada, the Maternal Newborn Child and Youth Strategic Clinical Network of Alberta Health, and the Owerko Centre of Alberta Children's Hospital Research Institute. C.P. is employed by the Public Health Agency of Canada.

## Supporting information


**Figure S1.** PRISMA flow diagram.

**Appendix S1.** Search strategies

**Table S1.** Risk of bias assessment for included studies

**Table S2.** Incidence studies

**Table S3.** Prevalence studies

**Table S4.** Prevalence of cervical dystonia 1985 to 2022
**Table S5.** Prevalence of blepharospasm, 1985 to 2022
**Table S6.** Prevalence of laryngeal dystonia, 1985 to 2022
**Table S7.** Prevalence of upper limb dystonia 1985 to 2022
**Table S8.** Prevalence of oromandibular dystonia 1985 to 2022
**Tables S9.** Prevalence of combined forms of dystonia, 1985 to 2022
