# Supplementary material for: The Prevalence of Idiopathic or Inherited Isolated Dystonia: A Systematic Review and Meta‐Analysis
Source: Mov Disord Clin Pract. 2022 Aug 24;9(7):860–8. doi: 10.1002/mdc3.13524 (PMC9547134; doi:10.1002/mdc3.13524)
Supplement: Supplementary file 2 — Appendix S1. Search strategies [file MDC3-9-860-s004.docx]

**Appendix 1. Search Strategy**

OVID

1. exp Dystonia/cl, di, ep, et, ge [Classification, Diagnosis, Epidemiology, Etiology, Genetics]

2. Dystonia*.tw,kf.

3. exp Dystonia Musculorum Deformans/cl, di, et, ge [Classification, Diagnosis, Etiology, Genetics]

4. Dystonia Musculorum Deformans*.tw,kf.

5. exp Dystonic Disorders/cl, di, ep, et, ge [Classification, Diagnosis, Epidemiology, Etiology, Genetics]

6. Dystonic Disorders*.tw,kf.

7. exp Blepharospasm/cl, di, ep, ge [Classification, Diagnosis, Epidemiology, Genetics]

8. Blepharospasm*.tw,kf.

9. exp Torticollis/cl, di, ep, ge [Classification, Diagnosis, Epidemiology, Genetics]

10. Torticollis*.tw,kf.

11. 1 or 2 or 3 or 4 or 5 or 6 or 7 or 8 or 9 or 10

12. exp Incidence/

13. incidence*.tw,kf.

14. exp Prevalence/

15. prevalence*.tw,kf.

16. exp Epidemiology/cl, di, mt, sn [Classification, Diagnosis, Methods, Statistics & Numerical Data]

17. 12 or 13 or 14 or 15 or 16

18. Dystonia/ep

19. Dystonia musculorum deformans/ep

20. Dystonic disorders/ep

21. Blepharospasm/ep

22. Torticollis/ep

23. 11 and 17

24. 18 or 19 or 20 or 21 or 22

25. 23 or 24

26. limit 25 to yr="2010 -Current"

27. limit 26 to animals

28. 26 not 27

EMBASE

1. exp dystonic disorder/di, ep, et [Diagnosis, Epidemiology, Etiology]

2. dystonic disorder*.tw,kw.

3. exp blepharospasm/di, ep, et [Diagnosis, Epidemiology, Etiology]

4. blepharospasm*.tw,kw.

5. exp torticollis/di, ep, et [Diagnosis, Epidemiology, Etiology]

6. torticollis*.tw,kw.

7. exp oromandibular dystonia/di, ep, et [Diagnosis, Epidemiology, Etiology]

8. oromandibular dystonia*.tw,kw.

9. exp torsion dystonia/di, ep, et [Diagnosis, Epidemiology, Etiology]

10. torsion dystonia*.tw,kw.

11. exp generalized dystonia/di, ep, et [Diagnosis, Epidemiology, Etiology]

12. generalized dystonia*.tw,kw.

13. exp segmental dystonia/di, ep, et [Diagnosis, Epidemiology, Etiology]

14. segmental dystonia*.tw,kw.

15. exp musician's dystonia/di, ep, et [Diagnosis, Epidemiology, Etiology]

16. musician's dystonia*.tw,kw.

17. exp focal dystonia/di, ep, et [Diagnosis, Epidemiology, Etiology]

18. focal dystonia*.tw,kw.

19. exp focal hand dystonia/di, ep, et [Diagnosis, Epidemiology, Etiology]

20. focal hand dystonia*.tw,kw.

21. exp dystonia/di, ep, et [Diagnosis, Epidemiology, Etiology]

22. dystonia*.tw,kw.

23. exp multifocal dystonia/di, ep, et [Diagnosis, Epidemiology, Etiology]

24. multifocal dystonia*.tw,kw.

25. exp cervical dystonia/di, ep, et [Diagnosis, Epidemiology, Etiology]

26. cervical dystonia*.tw,kw.

27. exp myoclonus dystonia/di, ep, et [Diagnosis, Epidemiology, Etiology]

28. myoclonus dystonia*.tw,kw.

29. exp paroxysmal dystonia/di, ep, et [Diagnosis, Epidemiology, Etiology]

30. paroxysmal dystonia*.tw,kw.

31. exp incidence/ep [Epidemiology]

32. incidence*.tw,kw.

33. exp prevalence/ep [Epidemiology]

34. prevalence*.tw,kw.

35. exp epidemiology/

36. epidemiology*.tw,kw.

37. 1 or 2 or 3 or 4 or 5 or 6 or 7 or 8 or 9 or 10 or 11 or 12 or 13 or 14 or 15 or 16 or 17 or 18 or 19 or 20 or 21 or 22 or 23 or 24 or 25 or 26 or 27 or 28 or 29 or 30

38. 31 or 32 or 33 or 34 or 35 or 36

39. 37 and 38

40. DYSTONIC DISORDERS/ep [Epidemiology]

41. BLEPHAROSPASM/ep [Epidemiology]

42. OROMANDIBULAR DYSTONIA/ep [Epidemiology]

43. TORTICOLLIS/ep [Epidemiology]

44. TORSION DYSTONIA/ep [Epidemiology]

45. GENERALIZED DYSTONIA/ep [Epidemiology]

46. SEGMENTAL DYSTONIA/ep [Epidemiology]

47. MUSICIANS DYSTONIA/ep [Epidemiology]

48. FOCAL DYSTONIA/ep [Epidemiology]

49. FOCAL HAND DYSTONIA/ep [Epidemiology]

50. DYSTONIA/ep [Epidemiology]

51. CERVICAL DYSTONIA/ep [Epidemiology]

52. MYOCLONUS DYSTONIA/ep [Epidemiology]

53. PAROXYSMAL DYSTONIA/ep [Epidemiology]

54. 40 or 41 or 42 or 44 or 45 or 46 or 47 or 48 or 49 or 50 or 51 or 52 or 53

55. 39 or 54

56. limit 55 to yr="2010 -Current"

57. limit 56 to animals

58. 56 not 57
