# Supplementary material for: The Prevalence of Idiopathic or Inherited Isolated Dystonia: A Systematic Review and Meta‐Analysis
Source: Mov Disord Clin Pract. 2022 Aug 24;9(7):860–8. doi: 10.1002/mdc3.13524 (PMC9547134; doi:10.1002/mdc3.13524)
Supplement: Supplementary file 3 — Table S1. Risk of bias assessment for included studies [file MDC3-9-860-s001.docx]

**Supplementary Materials Table I. Risk of Bias Assessment**

| Study | Is the target population clearly defined? | Was either of the following ascertainment method used? | | Is the response rate ≥70%? | Are non-responders clearly described? | Is the sample representative of the target population? | Were data collection methods standardized? | Were validated criteria used to assess for the presence/ absence of disease? | Are the estimates of prevalence and incidence given with confidence intervals and in detail by subgroup (if applicable)? | TOTAL QUALITY SCORE |
| --- | --- | --- | --- | --- | --- | --- | --- | --- | --- | --- |
|  |  | **Probability sampling** | **Entire population surveyed** |  |  |  |  |  |  |  |
| (Atehortua, Jaramillo et al. 2016) | 1 | 0 | 0 | 0 | 0 | 0 | 1 | 1 | 0 | 3 |
| (Badry, Abdelhamed et al. 2019) | 1 | 0 | 1 | 1 | 0 | 1 | 1 | 0 | 0 | 5 |
| (Bailey et al 2021) | 1 | 0 | 0 | 0 | 0 | 1 | 1 | 0 | 0 | 3 |
| (Bezerra, Novaretti et al. 2018) | 1 | 0 | 0 | 0 | 0 | 0 | 1 | 1 | 0 | 3 |
| (Bhidayasiri, Kaewwilai et al. 2011) | 1 | 0 | 0 | 0 | 0 | 0 | 1 | 1 | 1 | 4 |
| (Cubo, Doumbe et al. 2017) | 1 | 0 | 0 | 0 | 0 | 0 | 1 | 0 | 0 | 2 |
| (El-Tallawy, Farghaly et al. 2013) | 1 | 0 | 1 | 0 | 0 | 0 | 1 | 0 | 0 | 3 |
| (Fang, Xie et al. 2020) | 1 | 0 | 0 | 0 | 0 | 0 | 1 | 0 | 0 | 2 |
| (Hellberg, Alinder et al. 2019) | 1 | 0 | 0 | 0 | 0 | 0 | 1 | 0 | 1 | 3 |
| (Joensen 2016) | 1 | 0 | 0 | 0 | 0 | 0 | 1 | 1 | 1 | 4 |
| (LaHue, Albers et al. 2020) | 1 | 0 | 0 | 0 | 0 | 0 | 1 | 1 | 1 | 4 |
| (Louis, Eliasen et al. 2019) | 1 | 1 |  | 0 | 0 | 0 | 1 | 1 | 1 | 5 |
| (Ortiz, Scheperjans et al. 2018) | 1 | 0 | 0 | 0 | 0 | 0 | 1 | 1 | 1 | 4 |
| (Park, Damrauer et al. 2019) | 1 | 0 | 0 | 0 | 0 | 0 | 1 | 0 | 0 | 2 |
| (Sude and Nixdorf 2020) | 1 | 0 | 0 | 0 | 0 | 0 | 1 | 1 | 1 | 4 |
| (Sun, Tsai et al. 2018) | 1 | 0 | 0 | 0 | 0 | 0 | 1 | 1 | 1 | 4 |
| (Wang, Chen et al. 2016) | 1 | 0 | 0 | 0 | 0 | 0 | 1 | 1 | 1 | 4 |
| (Williams, McGovern et al. 2017) | 1 | 0 | 0 | 0 | 0 | 0 | 1 | 1 | 1 | 4 |
| (Yoshida 2021) | 1 | 0 | 0 | 0 | 0 | 0 | 1 | 1 | 1 | 4 |
