# Supplementary material for: The Prevalence of Idiopathic or Inherited Isolated Dystonia: A Systematic Review and Meta‐Analysis
Source: Mov Disord Clin Pract. 2022 Aug 24;9(7):860–8. doi: 10.1002/mdc3.13524 (PMC9547134; doi:10.1002/mdc3.13524)
Supplement: Supplementary file 4 — Table S2. Incidence studies [file MDC3-9-860-s003.docx]

**Supplementary Materials Table II. Dystonia Incidence Studies 2010 to 2022**

| Study, Date, Reference, Country | Population | Cases | Data Source | Diagnosis established by | Incidence Date | Overall Reported Incidence Rate | Subgroup Reported Incidence Rate |
| --- | --- | --- | --- | --- | --- | --- | --- |
| Bailey et al. 2021  Wales | People residing in Wales over the age of 20 years  47,557,077 person-years of observation | 41,660 new cases of adult onset dystonia reported during study period, 63% female, 37% male  Genetic torsion dystonia = 5  Idiopathic torsion dystonia = 39  Cervical dystonia = 26,563  Idiopathic orofacial dystonia = 24  Blepharospasm = 1181  Writer’s cramp = 41 | Wales Secure Anonymized Information Linkage (SAIL) Databank  Patients diagnosed with dystonia within the SAIL databank (were identified using a case ascertainment algorithm. Primary care data available for 80% of Welsh population; secondary care data available for 100% of Welsh population. | An individual was defined as having a diagnosis of dystonia if their GP or hospital record contained a ICD-10 code of G24.1, G24.2, G24.3, G24.4, G 24.5, G24.8, or G24.9, or a Read code of F136, F137, F137y, F138, F138z, F1360, F1382, 16A3, N135, N1350, N135z, F1380, F1383, F13B, F13C, Fyu24, Fyu2A, F13X or 1B22. Individuals diagnosed with a potential secondary cause of dystonia were excluded. | 1 January 1994-31 December 2017 | 87.6 per 100,000 per year | Incident rates (cases per 100,000 per year) by dystonia subgroup or sex not reported in publication |
| LaHue, Albers et al. 2020  USA | 15,489,433 person-years of observation | Cervical dystonia  n=200  Males 42  Females 158  <20=4  20-29=11  30-39=23  40-49=47  50-59=50  60-69=37  70-79=21  80+=7 | Clinical and administrative databases of Kaiser Permanente Northern California (KPNC)  healthcare system | Three stage case identification procedure used.  1. Identification of possible CD cases using a computerized search of electronic medical records using ICD-9 and diagnostic codes  2. Manual review and classification of utilization reports  3. Review by  movement disorder specialist | January 2003-December 2007 | 1.18 per 100,000 person-years (95% CI,0.35–2.0) Incidence standardized to the U.S. 2000 census | By sex  Male= 0.52 per 100,000 py (95% CI,0.1–0.9)  Female = 1.81 per 100,000 py (95% CI,1–2.5)  By age group  <20= 0.1 per 100,000 py (95% CI,0–0.6)  20-29= 0.6 per 100,000 py (95% CI,0–2.4)  30-39= 1.06 per 100,000 py (95% CI,0–3.2)  40-49= 1.91 per 100,000 py (95% CI,0–4.6)  50-59= 2.19 per 100,000 py (95% CI,0–5.2)  60-69= 2.57 per 100,000 py (95% CI,0–6.7)  70-79= 2.4 per 100,000 py (95% CI,0–7.5)  80+= 1.52 per 100,000 py (95% CI,0–7.2)  By ethnicity  African= 0.62 per 100,000 py (95% CI,0-2.51)  Asian= 0.46 per 100,000 py (95% CI,0–1.5)  White= 1.56 per 100,000 py (95% CI,0.36–2.76)  Native American= 0.93 per 100,000 py (95% CI,0–7.41) |
| Sun, Tsai et al. 2018  Taiwan | 18 years or older  1,000,000 | Blepharospasm  n=1325 | Longitudinal Health Insurance Database 2000, a subset of the National Health Insurance Research Database | ICD-9-CM code for blepharospasm, 333.81 | January 2000-December 2013 | Mean annual incidence was 10 per 100,000 person-years | By sex  Male= 7 per 100,000 py  Female= 12 per 100,000 py  By age group  18-29= 6 per 100,000 py  30-39= 12 per 100,000 py  40-49=16 per 100,000 py  50-59=19 per 100,000 py  60-69= 13 per 100,000 py  70-79= 8 per 100,000 py  80+=4 per 100,000 py |
| Yoshida et al. 2021  Japan | Residents of Kyoto Japan  N=1,465,701  7,000,000 person-years of observation | Oromandibular dystonia  N=84  Males 29  Females 55 | Residents of Kyoto diagnosed with oromandibular dystonia between January 2015 and December 2019 at the Department of Oral and Maxillofacial Surgery, Kyoto Medical Center | Diagnosis based on presence of at least 4 characteristic clinical features or findings of oromandibular dystonia. | January 2015- December 2019 | Mean annual incidence of idiopathic oromandibular dystonia was 1.2 per 100,000 person years (95%CI 0.68, 1.9) | Incidence by sex or age group not reported for idiopathic oromandibular dystonia |

Abbreviations: ICD, international classification of diseases; py, person-years
