# Supplementary material for: The Prevalence of Idiopathic or Inherited Isolated Dystonia: A Systematic Review and Meta‐Analysis
Source: Mov Disord Clin Pract. 2022 Aug 24;9(7):860–8. doi: 10.1002/mdc3.13524 (PMC9547134; doi:10.1002/mdc3.13524)
Supplement: Supplementary file 5 — Table S3. Prevalence studies [file MDC3-9-860-s005.docx]

**Supplementary Materials Table III. Dystonia Prevalence Studies 2010-2022**

| Study, Date, Reference, Country | Population | Cases | Data source | Diagnosis established by | Prevalence Date | Overall Reported Prevalence (per 100,000) | Subgroup Reported Prevalence (per 100,000) |
| --- | --- | --- | --- | --- | --- | --- | --- |
| Atehortua, Jaramillo et al. 2016  Colombia | Antioquia population =6,221,742 | n=874 (4,432 estimate made with log linear model)  Primary=692 Secondary=176 Plus=2  Not specified=4  Body distribution  Focal=660  Segmental=100  Generalized=63  Multifocal=38  Hemidystonia=7  Not specified=6  Cervical=325  Blepharospasm=138  Limb=136  Meige syndrome=43  Oromandibular=32  Laryngeal=29 | Cases of dystonia registered between June 2007 and June 2012 in 2 institutions of neurological referral in the city of Medellín and those  who went to the private medical practice of a neurologist who specializes in movement disorders. | Defined according to that which was recorded in the medical records in accordance with the criterion of the movement disorders specialist who evaluated each patient and following the phenomenological definition of dystonia and classification was performed  according to the criteria of Geyer and Bressman.  If data was insufficient the patients were contacted. | June 2007 - June 2012 | 712 per 1,000,000 (95% CI, 487.8–937) of dystonia (primary or secondary) | Not reported |
| Badry, Abdelhamed et al. 2019  Egypt | All age groups  Population in Al Qusier City = 33,285 subjects  Male/female distribution of population not given | Total=13  Primary=3  Secondary=10  Sex distribution:  Male 1  Females 2 | Population-based door-to-door study of the population of Al Qusier City, Red Sea Governorate, Egypt.  Phase I: Standardized screening questionnaire administered by neurologists  Phase II: Those who screened positive were invited to attend at Al Qusier General Hospital  where they were fully evaluated by three specialists of neurology each one separately. | Not reported. | Not reported | 39 per 100,000 for dystonia (primary or secondary) | By body distribution  Primary=9 per 100,000  Secondary=30 per 100,000 |
| Bailey et al. 2021  Wales | Population of Wales age 20 or older  N=2,721,833 | 32,662 with adult onset idiopathic dystonia | Wales Secure Anonymized Information Linkage (SAIL) Databank  Patients diagnosed with dystonia within the SAIL databank (were identified using a case ascertainment algorithm. Primary care data available for 80% of Welsh population; secondary care data available for 100% of Welsh population. | An individual was defined as having a diagnosis of dystonia if their GP or hospital record contained a ICD-10 code of G24.1, G24.2, G24.3, G24.4, G 24.5, G24.8, or G24.9, or a Read code of F136, F137, F137y, F138, F138z, F1360, F1382, 16A3, N135, N1350, N135z, F1380, F1383, F13B, F13C, Fyu24, Fyu2A, F13X or 1B22. Individuals diagnosed with a potential secondary cause of dystonia were excluded. | June 30 2017 | 1,200 per 100,000 | Not reported |
| Bezerra, Novaretti et al. 2018  Brazil | Above 17 years of age  n= 1,483,715 | Total= 289  235 patients used for characterization purposes; 91 male, 144 female  Primary=227  Dystonia plus=8  Dystonia Parkinsonism= 3  Dystonia myoclonus~~=~~5  Focal=152  Blepharospasm=60  Cervical Dystonia=51  Limb=28  Laryngeal~~=~~10  Oromandibular=3  Segmental=46  Meige=20  Craniocervical=13  Limb axial=6  Cranio-laryngo-cervical=4  Laryngo-cervical=2  Cranio-laryngeal=1  Multifocal=23  Generalized=14  Early onset generalized=9  Early onset multifocal=13  Early onset focal or segmental~~=~~21  Focal or segmental isolated adult=173 | All patients seen  in the Movement Disorders Section Ribeirao Preto School of  Medicine Hospital of the University of São Paulo | Diagnosis of primary dystonia or dystonia plus according to the classification of Fahn, Bressman, and  Marsden | March 2015 -February 2016 | 19.8 per 100,000 | Not reported |
| Bhidayasiri, Kaewwilai et al. 2011  Thailand | n=1,039,595 | Total= 207  Primary=141  58.2% female  Focal=149  Cervical=99  Writer’s cramp=21  Blepharospasm=12  Musician dystonia=4 | Database from the Chulalongkorn Comprehensive Movement Disorders Center.  Medical records  of the patients identified from the database were reviewed. | Primary dystonia  (1) dystonia was the sole abnormality  directly attributable to the condition, (2) there were no laboratory or imaging  abnormalities as well as no dramatic response to levodopa and (3) there was no  history of a known acquired or environmental cause of dystonia e for example,  neuroleptics exposure.  Secondary dystonia, specific causes were recorded  using the following categories: (1) tardive dystonia (meaning exposure to neuroleptic  or other dopamine receptor blocking agents for at least 3 months and the onset of  dystonia within 6 months after discontinuing the drug), (2) cerebrovascular disease,  (3) cerebral palsy, (4) cerebral trauma, (5) Wilson’s disease, (6) neurodegenerative  disorders and (7) other related causes | October 2005 - October 2010 | 19.9 per 100,000 (95% CI,17.2-22.6) | By body distribution  Primary=13.6 per 100,000 (95% CI, 17.2-22.6)  Focal=14.3 per 100,000 (95% CI, 12-16.6)  Cervical=9.5 per 100,000 (95% CI, 7.6-11.4)  Writer’s cramp=2.5 per 100,000 (95% CI,1.2-2.9)  Blepharospasm= 1.6 per 100,000 (95% CI,0.5-1.8) |
| Cubo, Doumbe et al. 2017  Cameroon | All ages  Inpatient/outpatient setting covered an urban population of 3,000,000 people | 18 | Patients with movement disorders were identified from a registry of neurological disorders in outpatient and inpatient settings from two urban public hospitals in Douala and two rural health care centers in Cameroon | Chart review; neurological diagnoses coded according to ICD-10, Dystonia disease G20 | May 2012 - May 2014 | 18 cases of dystonia identified from a population of 3,000,000 | Not reported |
| El-Tallawy, Farghaly et al. 2013  Egypt | All ages  n=33,285 | Cases not reported (no details on the forms of dystonia, or if dystonia cases included primary and/or secondary dystonia) | Door-to-Door survey.  All eligible inhabitants who had been living in Al Quseir  City for at least 6 months at the time of the interview were included in the survey. | Evaluation by the three neurologists. Criteria based  on the accepted definition and  diagnostic criteria given by the World Health Organization | July 1, 2009 - June 31, 2012 | 39.1 per 100,000 | Not reported |
| Fang, Xie et al. 2020  China | All ages  n=14,498,400 | Blepharospasm  =338  Males 98  Females 240 | Medical records from the Guangzhou Dystonia  Center in The First Affiliated Hospital, Sun Yat-sen  University | Unspecified | January 1999 - January 2018 | 2.33 per 100,000 | Not reported |
| Hellberg, Alinder et al. 2019  Sweden | All ages  Population of Sweden 9.64 million | 4,239 with primary dystonia  31.6% male  G24.1 Idiopathic familial dystonia 126  G24.2 Idiopathic nonfamilial dystonia 61  G24.3 Spasmodic torticollis 1742  G24.4 Idiopathic orofacial dystonia 140  G24.5 Blepharospasm 1133 | Swedish National  Patient Register (NPR)  Local data from Skåne University Hospital was also analyzed to calculate the estimated diagnostic uncertainty rate (20.2%). | All individuals who had received a diagnosis of non-drug-induced dystonia (ICD-10 G24.1–9) in the NPR at least twice over the 5 year study period.  Excluded individuals who also had ICD-10 diagnoses of Parkinson disease,  Parkinsonism, Huntington disease, Wilson disease, cerebral palsy, and  multiple sclerosis, or codes for external causes of morbidity attributed  to the diagnosis of dystonia. | 2009 - 2013 | Crude prevalence 44 per 100,000  Adjusted prevalence 35.1 per 100,000 (95% CI,34.0–36.3) | Not reported |
| Joensen 2016  Faroe Islands | All ages  n=48,100  0-19=12,621  20-49=19,143  50-59=5,772  60-69=5,223  70-79=5,341 | Primary focal dystonia=29  Males 10  Females 19  0-19=0  20-49=7  50-59=4  60-69=12  70-95=6  Cervical Dystonia=23  0-19=0  20-49=5  50-59=3  60-69=9  70-95=6  Writer’s cramp=4  Laryngeal=1  Oromandibular=1 | Medical charts review or medical interview from patients evaluated in the National Hospital of the Faroe Islands or private neurological practice, which constitutes all available neurological services on the Faroe Islands | Standard definition (Albanese MDS criteria) | January 1, 1994 - January 2014 | 602 per 1,000,000 (95% CI,395–873) | By age group  0-19=--  20-49=313 per 1,000,000 (95% CI, 104-679)  50-59=693 per 1,000,000 (95% C,I 173-1732)  60-69=2297 per 1,000,000 (95% CI, 1148-4020)  70-95=1122 per 1,000,000 (95% CI, 374-2334)  By body distribution  Cervical Dystonia= 478 per 1,000,000 (95% CI,332–728)  Writer’s cramp = 83 per 1,000,000 (95% CI,21-107)  Laryngeal=21 per 1,000,000 (95% CI, 0-84)  Oromandibular=21 per 1,000,000 (95% CI,0-84)  Primary focal dystonia  By sex:  Females =822 per million (95% CI, 476-1299)  Males= 400 per million (95% CI, 200-719) |
| Louis, Eliasen et al. 2019  Faroe islands | Population-based study  24,154 individuals aged ≥40 years living in the Faroe Islands, a  screening group comprised 3,000 Faroese individuals aged  ≥40 years was selected through random sampling.  Primary purpose of study was to determine the prevalence of tremor. | Of 227 participants with tremor who had an in-person evaluation, 77 total had dystonia  Thumb flexion dystonia= 75  Cervical dystonia=1  Segmental dystonia affecting cervical region and upper limb=1 | Multiphase procedure. Random sampling from the Faroese Population Registry to recruit 3000 participants in the first phase. 44.5% completed first phase; of these subsamples of 282 participants who completed first phase were invited to participate in an in-person clinical assessment. | The diagnosis of dystonia was made using published diagnostic  criteria (sustained or intermittent muscle contractions causing abnormal, often repetitive, movements, postures, or both) | August 2016- December 2017 | 33.92 per 100,000  (95% CI,27.73–40.11) | By body distribution  Cervical Dystonia + segmental Dystonia=  (0.88%, 95% CI,0.33-2.09%)  Thumb Flexion Dystonia=  33.33%, 95% CI 27.17–39.49% |
| Ortiz, Scheperjans et al. 2018  Finland | Average population over 20 years 2007–2016: 1,580,758 | 1316 Adult Onset Isolated Idiopathic or hereditary dystonia patients  Male to female ratio 1:2.7 | National Care Register Data and patient records | ICD-10 codes  G24 dystonia, G24.1 idiopathic familial dystonia, G24.2 idiopathic non-familial dystonia, G24.3 spasmodic torticollis, G24.4 idiopathic orofacial dystonia, G24.5 blepharospasm, G24.8 other dystonia, G24.9 dystonia, unspecified  Medical records were checked for validation for patients residing in two provinces. | 2007 - 2016 | 405 per 1,000,000 ± 46 | By body distribution  Focal cervical=304 per 1,000,000 ± 34  Focal upper cranial =26 per 1,000,000 ± 5  Focal upper limb= per 1,000,000 15 ± 7  Focal laryngeal=12 per 1,000,000 ± 5  Focal lower cranial=5 per 1,000,000 ± 2  Focal lower limb=1 per 1,000,000 ± 1  Focal axial 1 per=1,000,000 ± 1  Segmental=33 per 1,000,000 ± 5  Multifocal=6 per 1,000,000 ± 2  Generalized=2 per 1,000,000 ± 1  By age group (all dystonia)  20–39 years=91 per 1,000,000 ± 6  40–59 years=650 per 1,000,000 ± 37  60–79 years=889 per 1,000,000 ± 146  over 80 years=415 per 1,000,000 ± 83 |
| Park, Damrauer et al. 2019  USA | 11,451 individuals who have undergone  whole-exome sequencing | DYT1 Carriers | Exome sequencing data from the Genome Aggregation Database (gnomAD) and Penn Medicine Biobank (PMBB) | Diagnostic codes from the EMR of all PMBB participants with  exome data for ICD-9 codes 333.6, 333.7, and 333.8 to correlate the p.D216H polymorphism with dystonia. | Not reported | gnomAD 17.6 carriers/100,000  PMBB 26.2 carriers/100,000 | Not reported |
| Sude and Nixdorf 2020  USA | Above 18 years of age  n=3572 new patients evaluated at TMD, Orofacial Pain, and Dental Sleep Medicine  Clinic at the University of Minnesota from 2012 to 2017 | Oromandibular Dystonia=6 | Registry and charts of patients evaluated  in the TMD, Orofacial Pain, and Dental Sleep Medicine  Clinic at the University of Minnesota | ICD-9 diagnostic code 333.82 and ICD-10 code G24.4  Charts of eligible patients were  reviewed to confirm the accuracy of the documented diagnosis. | October 2012 - December 2017 | 170 per 100,000 persons (95% CI, 70-390) evaluated at TMD, Orofacial Pain, and Dental Sleep Medicine  Clinic at the University of Minnesota | Not reported |
| Wang, Chen et al. 2016  China | >26 years  N= 54,938,000 | Late-onset primary dystonia  n=1,481  Males 486  Females 995  Focal=1134  Segmental=301  Multifocal=24  Generalized=22  Blepharospasm =640  Cervical dystonia= 416 Limb dystonia=38  Oromandibular= 33  Laryngeal=7 | Database and records from the movement disorder and botulinum toxin clinic at Sir Run Run Shaw Hospital | Unspecified | January 2009 - December 2013 | 27.0 per 1,000,000 (95 % CI,25.6–28.3) | Not reported |
| Williams, McGovern et al. 2017  Ireland | Patients 20 years of age or older  2011 Ireland census  3,325,821 | Adult Onset Isolated Dystonia n=592  Cervical Dystonia=410  Blepharospasm=102  Focal hand  Dystonia=39  Spasmodic  Dysphonia=18  Musician dystonia=17  Oromandibular=6 | Database of patients attending the dystonia botulinum toxin clinic in St Vincent’s University Hospital, Dublin. Also from 6 neurology and 3 ophthalmology clinics in Ireland.  Assessment by a movement disorders specialist | Patients aged 20 years or older, diagnosed with adult onset isolated focal dystonia and phenotyped by a neurologist with expertise in movement disorders. Those with secondary dystonia, primary with onset prior to 20 years of age or with DYT1 dystonia were excluded. | Prevalence date was 31 December 2014 | 17.8 per  100 000 (95% CI, 16.4-19.2) | By sex  Males= 9.3 per  100 000  Females= 21 per  100 000  Supplementary materials provide point estimates by gender divided by group ages, but not a total (men +women) by age nor body distribution. |
| Yoshida 2021  Japan | Residents of Kyoto Japan  N=1,465,701  Males 694,247  Females 771,454 | Idiopathic oromandibular dystonia = 84  Males 55  Females 37 | Residents of Kyoto diagnosed with oromandibular dystonia between January 2015 and December 2019 at the Department of Oral and Maxillofacial Surgery, Kyoto Medical Center | Diagnosis based on presence of at least 4 characteristic clinical features or findings of oromandibular dystonia. | Prevalence date was January 1 2020 | 5.7 per 100,000 (95% CI 4.6-7.1) | By gender  Males= 4.18 per 100,000  Females= 7.3 per 100,000 |

Abbreviations: GP, general practitioner; ICD, international classification of diseases
