# Supplementary material for: The Prevalence of Idiopathic or Inherited Isolated Dystonia: A Systematic Review and Meta‐Analysis
Source: Mov Disord Clin Pract. 2022 Aug 24;9(7):860–8. doi: 10.1002/mdc3.13524 (PMC9547134; doi:10.1002/mdc3.13524)
Supplement: Supplementary file 6 — Table S4. Prevalence of cervical dystonia 1985 to 2022 Table S5. Prevalence of blepharospasm, 1985 to 2022 Table S6. Prevalence of laryngeal dystonia, 1985 to 2022 Table S7. Prevalence of upper limb dystonia 1985 to 2022 Table S8. Prevalence of oromandibular dystonia 1985 to 2022 Tables S9. Prevalence of combined forms of dystonia, 1985 to 2022 [file MDC3-9-860-s002.docx]

**Supplementary Table IV Prevalence of Cervical Dystonia 1985 to 2022**

| **Location** | **Study** | **Cases** | **Sample** | **Prevalence per 100,000** | **95% CI** |
| --- | --- | --- | --- | --- | --- |
| South America (Brazil) | (Bezerra, Novaretti et al. 2018) | 51 | 1,483,715 | 3.44 | 2.61-4.52 |
| Asia (China) | (Wang, Chen et al. 2016) | 416 | 54,938,000 | 0.76 | 0.69-0.83 |
| Asia (Thailand) | (Bhidayasiri, Kaewwilai et al. 2011) | 99 | 1,039,595 | 9.52 | 7.82-11.60 |
| Asia (Japan) | Fukuda et al. 2006 | 5 | 247,973 | 2.02 | 0.84-4.84 |
| Asia (Japan) | Matsumoto et al. 2003 | 34 | 1,459,130 | 2.33 | 1.67-3.26 |
| Asia (Japan) | Sugawara et al. 2006 | 33 | 1,166,967 | 2.83 | 2.01-4.00 |
| ***Subgroup analysis Asia I2=0, Q=1.6*** | | | | **2.50** | **0.71-8.75** |
| Europe (Sweden) | (Hellberg, Alinder et al. 2019) | 1742 | 9,640,000 | 18.07 | 17.24-18.94 |
| Europe (Faroe Islands) | (Joensen 2016) | 23 | 48,100 | 47.81 | 31.78-71.95 |
| Europe (Faroe Islands) | (Louis, Eliasen et al. 2019) | 1 | 24,154 | 4.14 | 0.58-29.38 |
| Europe (Finland) | (Ortiz, Scheperjans et al. 2018) | 589 | 1,580,758 | 37.30 | 34.37-40.40 |
| Europe (Ireland) | (Williams, McGovern et al. 2017) | 410 | 3,325,821 | 8.94 | 8.11-9.84 |
| Europe (Iceland) | Asgeirsson et al. 2006 | 33 | 288,201 | 11.45 | 8.14-16.11 |
| Europe | ESDE, 2000 | 330 | 57,92,937 | 5.70 | 5.11-6.45 |
| Europe (Germany) | Konkiewitz et al, 2002 | 72 | 1,322,883 | 5.44 | 4.32-6.86 |
| Europe (Norway) | Le at al. 2003 | 66 | 508,726 | 12.97 | 10.19-16.51 |
| Europe (Italy) | Papantonio et al. 2009 | 24 | 541,653 | 4.44 | 2.97-6.61 |
| Europe (Serbia) | Pekmezovic et al. 2003 | 72 | 1,602,226 | 4.49 | 3.57-5.66 |
| ***Subgroup analysis Europe I2=30.1, Q=12.9*** | | | | **11.38** | **7.35-17.61** |
| **Total I2=0, Q=15** | | | | **6.62** | **3.55-12.33** |

**Supplementary Table V Prevalence of Blepharospasm, 1985-2022**

| **Location** | **Study** | **Cases** | **Sample** | **Prevalence per 100,000** | **95% CI** |
| --- | --- | --- | --- | --- | --- |
| South America (Brazil) | (Bezerra, Novaretti et al. 2018) | 60 | 1,483,715 | 4.04 | 3.14-5.21 |
| Asia (China) | (Wang, Chen et al. 2016) | 640 | 54,938,000 | 1.16 | 1.08-1.26 |
| Asia (Thailand) | (Bhidayasiri, Kaewwilai et al. 2011) | 12 | 1,039,595 | 1.2 | 0.7-2 |
| Asia (China) | (Fang, Xie et al. 2020) | 338 | 14,498,400 | 2.33 | 2.10-2.59 |
| Asia (Japan) | Matsumoto et al. 2003 | 49 | 1,459,130 | 3.36 | 2.54-4.44 |
| Asia (Japan) | Sugawara et al. 2006 | 122 | 1,166,967 | 10.45 | 8.75-12.48 |
| ***Subgroup analysis Asia* *I^2^=4.3, Q=4.2*** | | | | **2.59** | **1.19-5.66** |
| Europe (Sweden) | (Hellberg, Alinder et al. 2019) | 1,133 | 9,640,000 | 11.75 | 11.09-12.46 |
| Europe (Finland) | (Ortiz, Scheperjans et al. 2018) | 47 | 1,580,758 | 2.97 | 2.23-3.96 |
| Europe (Ireland) | (Williams, McGovern et al. 2017) | 102 | 3,325,821 | 3.07 | 2.52-3.72 |
| Europe (Iceland) | Asgeirsson et al. 2006 | 9 | 288,201 | 3.12 | 1.62-6.00 |
| Europe (Italy) | Cossu et al 2006 | 53 | 1,652,332 | 3.21 | 2.45-4.20 |
| Europe (Italy) | Defazio et al 2001 | 9 | 67,606 | 13.31 | 6.93-25.58 |
| Europe | ESDE, 2000 | 208 | 5,792,937 | 3.59 | 3.13-4.11 |
| Europe (Germany) | Konkiewitz et al, 2002 | 41 | 1,322,883 | 3.10 | 2.29-4.21 |
| Europe (Norway) | Le at al. 2003 | 24 | 508,726 | 4.72 | 3.16-7.04 |
| Europe (Italy) | Papantonio et al. 2009 | 32 | 541,653 | 5.91 | 4.18-8.35 |
| Europe (Serbia) | Pekmezovic et al. 2003 | 23 | 1,602,226 | 1.44 | 0.95-2.16 |
| ***Subgroup analysis Europe* *I^2^=0, Q=5.7*** | | | | **4.14** | **2.51-6.82** |
| **Total I^2^=0, Q=7.1** | | | | **3.60** | **2.15-6.00** |

**Supplementary Table VI Prevalence of Laryngeal Dystonia, 1985 to 2022**

| **Location** | **Study** | **Cases** | **Sample** | **Prevalence per 100,000** | **95% CI** |
| --- | --- | --- | --- | --- | --- |
| South America (Brazil) | (Bezerra, Novaretti et al. 2018) | 10 | 1,483,715 | 0.67 | 0.36-1.25 |
| Asia (China) | (Wang, Chen et al. 2016) | 7 | 54,938,000 | 0.01 | 0.01-0.03 |
| Europe (Faroe Islands) | (Joensen 2016) | 1 | 48,100 | 2.08 | 0.29-14.76 |
| Europe (Finland) | (Ortiz, Scheperjans et al. 2018) | 22 | 1,580,758 | 1.39 | 0.92-2.11 |
| Europe (Ireland) | (Williams, McGovern et al. 2017) | 18 | 3,325,821 | 0.54 | 0.34-0.86 |
| Europe (Iceland) | Asgeirsson et al. 2006 | 17 | 288,201 | 5.90 | 3.67-9.49 |
| Europe (Germany) | Konkiewitz et al. 2002 | 13 | 1,322,883 | 0.98 | 0.57-1.69 |
| Europe (Norway) | Le et al., 2003 | 14 | 5,792,937 | 2.75 | 1.63-4.65 |
| Europe | ESDE 2000 | 39 | 5,792,937 | 0.67 | 0.49-0.92 |
| Europe (Serbia) | Pekmezovic 2003 | 13 | 1,602,226 | 0.81 | 0.47-1.40 |
| ***Subgroup analysis Europe I^2^=0 Q=6.3*** | | | | **1.34** | **0.73-2.45** |
| **Total I^2^=35.8, Q=14.0** | | | | **0.79** | **0.35-1.79** |

**Supplementary Table VII Prevalence of Upper Limb Dystonia 1985-2022**

| **Location** | **Study** | **Cases** | **Sample** | **Prevalence per 100,000** | **95% CI** |
| --- | --- | --- | --- | --- | --- |
| South America (Brazil) | (Bezerra, Novaretti et al. 2018) | 28 | 1,483,715 | 1.89 | 1.30-2.73 |
| Asia (China) | (Wang, Chen et al. 2016) | 38 | 54,938,000 | 0.07 | 0.05-0.09 |
| Asia (Thailand) | (Bhidayasiri, Kaewwilai et al. 2011) | 21 | 1,039,595 | 2.02 | 1.32-3.10 |
| Asia (Japan) | Suguwara | 13 | 1,166,967 | 1.11 | 0.65-1.92 |
| Asia (Japan) | Matsumoto et al. 2003 | 23 | 1,459,130 | 1.58 | 1.05-2.37 |
| Asia (Japan) | Fukuda et al., 2006 | 11 | 247,973 | 4.44 | 2.46-8.01 |
| ***Subgroup analysis Asia I^2^=0 Q=2.9*** | | | | **1.01** | **0.20-5.17** |
| Europe (Faroe Islands) | (Joensen 2016) | 4 | 48,100 | 8.32 | 3.12-22.16 |
| Europe (Finland) | (Ortiz, Scheperjans et al. 2018) | 31 | 1,580,758 | 1.96 | 1.38-2.79 |
| Europe (Ireland) | (Williams, McGovern et al. 2017) | 39 | 3,325,821 | 1.17 | 0.86-1.60 |
| Europe (Iceland) | Asgeirsson et al. 2006 | 23 | 288,201 | 7.98 | 5.30-12.01 |
| Europe | ESDE, 2000 | 79 | 5,792,937 | 1.36 | 1.09-1.70 |
| Europe (Norway) | Le at al. 2003 | 12 | 508,726 | 2.36 | 1.34-4.15 |
| Europe (Serbia) | Pekmezovic et al. 2003 | 23 | 1,602,226 | 1.44 | 0.95-2.16 |
| ***Subgroup analysis Europe I^2^=23.8, Q=7.8*** | | | | **2.39** | **1.43-4.01** |
| **Total I^2^=0, Q=11.1** | | | | **1.71** | **0.88-3.34** |

**Supplementary Table VIII Prevalence of Oromandibular Dystonia 1985-2022**

| **Location** | **Study** | **Cases** | **Sample** | **Prevalence per 100,000** | **95% CI** |
| --- | --- | --- | --- | --- | --- |
| South America (Brazil) | (Bezerra, Novaretti et al. 2018) | 3 | 1,483,715 | 0.20 | 0.07-0.63 |
| Asia (China) | (Wang, Chen et al. 2016) | 33 | 54,938,000 | 0.06 | 0.04-0.08 |
| Asia (Japan) | (Yoshida 2021) | 84 | 1,465,701 | 5.73 | 4.63-7.10 |
| Asia (Japan) | Matsumoto et al. 2003 | 11 | 1,459,130 | 0.75 | 0.42-1.36 |
| Asia (Japan) | Nakashima et al 1995 | 1 | 244,935 | 0.41 | 0.05-2.90 |
| ***Subgroup analysis Asia I^2^=0, Q=1.37*** | | | | **0.58** | **0.04-9.08** |
| Europe (Finland) | (Ortiz, Scheperjans et al. 2018) | 9 | 1,580,758 | 0.57 | 0.30-1.09 |
| Europe (Ireland) | (Williams, McGovern et al. 2017) | 6 | 3,325,821 | 0.18 | 0.08-0.40 |
| Europe (Iceland) | Asgeirsson et al. 2006 | 8 | 288,201 | 2.77 | 1.39-5.55 |
| Europe | ESDE, 2000 | 5 | 5,792,937 | 0.08 | 0.04-0.21 |
| Europe (Norway) | Le at al. 2003 | 4 | 508,726 | 0.79 | 0.30-2.09 |
| Europe (Italy) | Papantonio et al. 2009 | 1 | 541,653 | 0.18 | 0.03-1.31 |
| Europe (Sweden) | Hellberg 2019 | 140 | 9,640,000 | 1.45 | 1.23-1.71 |
| Europe (Faroe Islands) | Joensen 2016 | 1 | 48,100 | 2.08 | 0.29-14.76 |
| ***Subgroup analysis Europe I^2^=10.0, Q=7.8*** | | | | **0.58** | **0.25-1.33** |
| **Total I^2^=0, Q=8.0** | | | | **0.53** | **0.21-1.36** |

| **Location** | **Study** | **Cases** | **Sample** | **Prevalence per 100,000** | **95% CI** |
| --- | --- | --- | --- | --- | --- |
| Egypt (Africa) | Badry 2019 | 3 | 33,285 | 9.01 | 2.91-27.94 |
| South America (Brazil) | (Bezerra, Novaretti et al. 2018) | 227 | 1,483,715 |  |  |
| Asia (China) | (Wang, Chen et al. 2016) | 1,481 | 54,938,000 | 2.70 | 2.56-2.84 |
| Asia (Thailand) | (Bhidayasiri, Kaewwilai et al. 2011) | 141 | 1,039,595 | 13.56 | 11.50-16.00 |
| Asia (Japan) | Matsumoto et al. 2003 | 147 | 1,459,130 | 10.07 | 8.57-11.84 |
| Asia (Japan) | Sugawara et al. 2006 | 177 | 1,166,967 | 15.17 | 13.09-17.57 |
| ***Subgroup analysis Asia I2=0, Q=1.7*** | | | | **8.63** | **3.05-24.43** |
| Europe (Sweden) | (Hellberg, Alinder et al. 2019) | 4,974 | 9,640,000 | 51.60 | 50.18-53.05 |
| Europe (Wales) | Bailey 2021 | 32,662 | 2,721,833 | 1200.00 | 1187.13-1213.00 |
| Europe (Faroe Islands) | (Joensen 2016) | 29 | 48,100 | 60.29 | 41.90-86.75 |
| Europe (Finland) | (Ortiz, Scheperjans et al. 2018) | 1,316 | 1,580,758 | 83.25 | 78.87-87.87 |
| Europe (Ireland) | (Williams, McGovern et al. 2017) | 592 | 3,325,821 | 17.80 | 16.42-19.29 |
| Europe (Iceland) | Asgeirsson et al. 2006 | 107 | 288,201 | 37.13 | 30.72-44.87 |
| Europe (Germany) | Konkiewitz et al. 2002 | 188 | 1,322,883 | 14.21 | 12.32-16.40 |
| Europe | ESDE 2000 | 879 | 5,792,937 | 15.17 | 14.20-16.21 |
| ***Subgroup analysis Europe I2=0, Q=2.7*** | | | | **50.87** | **10.27-251.56** |
| **Total I2=0 Q=3.6** | | | | **25.38** | **6.21-103.67** |

**Supplementary Table IX Prevalence of Combined Forms of Dystonia, 1985 to 2022**
